# Exposure to high concentrations of inspired oxygen does not worsen lung injury after cardiac arrest

**DOI:** 10.1186/s13054-015-0824-x

**Published:** 2015-03-10

**Authors:** Jonathan Elmer, Bo Wang, Samer Melhem, Raghevesh Pullalarevu, Nishit Vaghasia, Jaya Buddineni, Bedda L Rosario, Ankur A Doshi, Clifton W Callaway, Cameron Dezfulian, on behalf of the University of Pittsburgh Post-Cardiac Arrest Service (PCAS)

**Affiliations:** Safar Center for Resuscitation Research, University of Pittsburgh School of Medicine, 100 Hill Building, 3434 Fifth Avenue, Pittsburgh, PA 15260 USA; Department of Critical Care Medicine, University of Pittsburgh School of Medicine, Pittsburgh, USA; Department of Emergency Medicine, University of Pittsburgh School of Medicine, Pittsburgh, USA; Department of Anesthesiology, New York University Langone Medical Center, Pittsburgh, USA; Department of Internal Medicine, University of Pittsburgh Medical Center Mercy Hospital, Pittsburgh, USA; Department of Epidemiology, Graduate School of Public Health, University of Pittsburgh, Pittsburgh, USA; Vascular Medicine Institute, University of Pittsburgh School of Medicine, Pittsburgh, USA

## Abstract

**Introduction:**

Post-cardiac arrest patients are often exposed to 100% oxygen during cardiopulmonary resuscitation and the early post-arrest period. It is unclear whether this contributes to development of pulmonary dysfunction or other patient outcomes.

**Methods:**

We performed a retrospective cohort study including post-arrest patients who survived and were mechanically ventilated at least 24 hours after return of spontaneous circulation. Our primary exposure of interest was inspired oxygen, which we operationalized by calculating the area under the curve of the fraction of inspired oxygen (FiO_2_AUC) for each patient over 24 hours. We collected baseline demographic, cardiovascular, pulmonary and cardiac arrest-specific covariates. Our main outcomes were change in the respiratory subscale of the Sequential Organ Failure Assessment score (SOFA-R) and change in dynamic pulmonary compliance from baseline to 48 hours. Secondary outcomes were survival to hospital discharge and Cerebral Performance Category at discharge.

**Results:**

We included 170 patients. The first partial pressure of arterial oxygen (PaO2):FiO2 ratio was 241 ± 137, and 85% of patients had pulmonary failure and 55% had cardiovascular failure at presentation. Higher FiO_2_AUC was not associated with change in SOFA-R score or dynamic pulmonary compliance from baseline to 48 hours. However, higher FiO_2_AUC was associated with decreased survival to hospital discharge and worse neurological outcomes. This was driven by a 50% decrease in survival in the highest quartile of FiO_2_AUC compared to other quartiles (odds ratio for survival in the highest quartile compared to the lowest three quartiles 0.32 (95% confidence interval 0.13 to 0.79), *P* = 0.003).

**Conclusions:**

Higher exposure to inhaled oxygen in the first 24 hours after cardiac arrest was not associated with deterioration in gas exchange or pulmonary compliance after cardiac arrest, but was associated with decreased survival and worse neurological outcomes.

**Electronic supplementary material:**

The online version of this article (doi:10.1186/s13054-015-0824-x) contains supplementary material, which is available to authorized users.

## Introduction

Over 500,000 Americans suffer a cardiac arrest (CA) annually [1]. Among those with return of spontaneous circulation (ROSC), 50 to 60% do not survive to hospital discharge [2,3]. The post-arrest syndrome develops commonly after CA, and is characterized by multiple organ dysfunction secondary to global ischemia-reperfusion injury and a systemic inflammatory response [4-6]. Pulmonary dysfunction and the acute respiratory distress syndrome (ARDS) are components of this process.

Observational studies have associated higher arterial oxygen concentration (hyperoxia) with worsened clinical outcomes [7-10]. Authors of these studies have hypothesized that this effect is mediated through worsening of secondary brain injury by increased oxidative stress and free radical formation, but other possible explanations for this association have not been previously explored. In the lung, oxygen exposure may to lead to ARDS through free-radical formation [11,12], and although isolated respiratory failure is an uncommon proximate cause of death after CA, lung dysfunction is common and may worsen patient outcomes [13]. Preclinical data suggest that high concentrations of oxygen cause pulmonary endothelial apoptotic cell death and worsened lung injury [14-17]. However, there is an important interaction between tidal volume (that is, stretch-induced lung injury) and oxygen toxicity that may explain this observation [18,19]. Indeed, early clinical reports of pulmonary oxygen toxicity were conducted when open-lung ventilation with high tidal volumes was standard practice [20-22].

CA patients are often exposed to 100% oxygen during cardiopulmonary resuscitation (CPR) and early after ROSC [10]. Thus, this cohort is theoretically at risk for oxygen-induced lung toxicity, which might help explain the association between hyperoxia and patient outcomes. Respiratory failure based on a higher sequential organ failure assessment (SOFA) score has been associated with increased mortality in two studies of CA outcomes [23,24]. We analyzed data from a high-resolution CA database to test the hypothesis that cumulative dose of inhaled oxygen to which patients are exposed after CA is associated with deterioration in lung function in the first 48 h after ROSC. Second, we tested the association between inhaled oxygen exposure and clinically relevant patient outcomes including survival to hospital discharge and neurological outcome.

## Materials and methods

### Patients and setting

We analyzed a prospective database of consecutive in- and out-of-hospital CA patients cared for at UPMC Presbyterian hospital by the Post-Cardiac Arrest Service (PCAS). UPMC Presbyterian is a 798-bed tertiary care center with approximately 54,000 emergency department visits annually and 150 ICU beds. The PCAS treats over 350 survivors of CA annually and is part of local efforts to regionalize and improve CA care. We have implemented hospital-wide protocols to standardize and improve post-CA care [25].

We included CA patients presenting between October 2008 (when electronic medical records were implemented system-wide, permitting recording of vital signs and ventilator data) and April 2010, who had a CA with ROSC, survived and were mechanically ventilated for a least 24 h. We included both in- and out-of-hospital CA patients because 1) this reflects the true heterogeneity encountered in clinical practice, 2) we hypothesized that post-arrest organ dysfunction would be primarily dependent on ischemia and reperfusion injury rather than pathology present antecedent to the arrest, and 3) we have previously demonstrated that the affect of cardiopulmonary dysfunction on patient outcomes is stable across arrest location [23]. We excluded patients if the time of ROSC was unknown, if vital signs and vasopressor requirements were not recorded within 6 h of ROSC, or if arterial blood gas (ABG) or ventilator data were not recorded within 4 h of ROSC (we have previously reported that this is the timeframe during which patients are most often exposed to high fraction of inspired oxygen (FiO_2_) levels [10]). We further excluded patients managed with extracorporeal membrane oxygenation. The University of Pittsburgh Institutional Review Board approved all aspects of this study with a waiver of informed consent.

### Primary exposure and covariates

Our primary exposure of interest was inhaled oxygen. To quantify this exposure, we calculated the area under the curve (AUC) of the FiO_2_ (FiO_2_AUC) for each patient as follows. First, we recorded FiO_2_ values hourly for 24 h after ROSC. Based on our local practice, we assumed all patients were ventilated with an FiO_2_ of 1.0 during their initial resuscitation until the first recorded value [10]. Next, we summed these hourly values to derive the FiO_2_AUC. This FiO_2_AUC could theoretically range from 5 (24 h on room air at FiO_2_ = 0.21) to 24 (24 h on FiO_2_ = 1.0).

We abstracted demographic and basic clinical information from our prospective CA registry, including subject age, gender, location of arrest (out-of-hospital versus in-hospital), initial arrest rhythm (ventricular tachycardia or fibrillation (VT/VF) versus pulseless electrical activity (PEA) or asystole) and use of hypothermia. Additionally we abstracted each subject’s Pittsburgh CA Category (PCAC). The PCAC is a clinical prediction tool that stratifies post-arrest patients by their risk of subsequent death or neurological deterioration based on clinical characteristics during the first 6 h after ROSC [23]. This tool divides patients into four categories that are strongly predictive of survival and functional outcome.

We assessed baseline post-arrest pulmonary dysfunction by recording each patient’s initial partial pressure of arterial oxygen (PaO_2_):FiO_2_ (P:F) ratio and dynamic pulmonary compliance, which we calculated as equal to tidal volume/(peak inspiratory pressure minus positive end-expiratory pressure (PEEP)). Additionally, we calculated the respiratory subscale of the SOFA scale (SOFA-R) [26]. To quantify the degree of post-arrest cardiovascular dysfunction, we calculated the baseline cardiovascular subscale of the SOFA score (SOFA-CV), cumulative vasopressor index (CVI), and shock index (mean arterial pressure/heart rate). The SOFA score is a validated measure of organ dysfunction commonly used in critical care research [26]. The respiratory subscale assigns 0 to 4 points based on the P:F ratio and requirement for mechanical ventilation and the cardiovascular subscale assigns 0 to 4 points based on the presence of hypotension and vasoactive medication requirement. The CVI is a method of standardizing vasopressor dosing [26,27]. We calculated the CVI from the highest vasopressor dose needed to achieve a mean arterial pressure (MAP) >70 mmHg within the first 6 h after ROSC.

### Missing data

The baseline data necessary to calculate each patient’s baseline SOFA score were unavailable in the first 6 h in a small number of cases. In this situation, we used data within 12 h of the time of interest. When PaO_2_ data were unavailable even within 12 h of a given time point (3.2% of cases), we calculated SOFA-R using peripheral oxygen saturation (SpO_2_) which is recorded hourly based on the following table, which we developed on the basis of a previously validated SpO_2_:FiO_2_ estimation for P:F ratio [28].

### Outcomes

The primary outcomes of interest were the change in SOFA-R and dynamic pulmonary compliance over the first 48 h after admission, and second, the changes in SOFA-R and dynamic pulmonary compliance from 0 to 24 h and 0 to 72 h. As lung injury develops over hours-to-days after the initial inflammatory or injurious insult, we chose these time points to ensure we fully captured the sequelae of the initial exposure [29]. Additional secondary outcomes of interest were 1) survival to hospital discharge, and 2) neurological outcomes, which we assessed by measuring the Pittsburgh cerebral performance category (CPC) at hospital discharge.

### Statistical analysis

We used Stata Version 13.1 (StataCorp, College Station, TX, USA) for our analyses. We used linear regression to test the association between baseline characteristics and FiO_2_AUC. Next, we explored the association between FiO_2_AUC and change in SOFA-R (using ordered logistic regression) and change in dynamic pulmonary compliance (using linear regression). Finally, we investigated the association between FiO_2_AUC and survival (using unadjusted and multiple logistic regression) and neurological outcome (using unadjusted and multiple ordered logistic regression). In the adjusted models we included only variables with unadjusted associations having outcomes with *P*-values <0.2, to avoid over-fitting. We confirmed the validity of the proportional odds assumption for ordered logistic regression test procedures. As a *post hoc* analysis, to determine whether baseline pulmonary dysfunction or change in pulmonary dysfunction differed between the in-hospital and out-of-hospital arrest populations, we stratified patients by arrest location and compared their baseline SOFA-R, dynamic pulmonary compliance and the change in these values from baseline to 72 h and 24 to 72 h using Fisher’s exact tests and *t*-tests as appropriate.

To evaluate the possibility of a threshold effect for an association between oxygen exposure and clinical outcomes, we divided the population into quartiles based on FiO_2_AUC and compared survival and neurological outcome between these quartiles. Finally, to address the possibility of confounding by indication (that is, severity of illness may lead both to exposure to higher FiO_2_AUC and to worse outcomes in the absence of causality), we performed a propensity-adjusted analysis. We generated a saturated propensity score incorporating all baseline covariates to model the propensity for exposure to a higher quartile of FiO_2_AUC given baseline clinical characteristics, and then calculated the inverse probability of treatment weight (IPTW) using the inverse of the propensity score and a weight to reflect the sample size of each treatment group [30,31]. After checking for and removing any extreme outlier IPTW values, we constructed adjusted models using the IPTW to test for an independent association between FiO_2_AUC quartile and change in SOFA-R and dynamic pulmonary compliance from 0 to 48 h, as well as overall survival.

## Results

### Cohort characteristics

Our initial registry query yielded 187 CA patients, of whom 17 were transferred from outlying facilities late in their post-arrest course and therefore excluded for missing post-ROSC ABG, vital sign or ventilator data based on our exclusion criteria, leaving 170 patients for analysis. Mean age was 60 ± 6 years and 52% were male (Table 1). The baseline PaO_2_:FiO_2_ ratio was 241 ± 137, and quantified using SOFA subscales, 85% of patients had baseline pulmonary failure while 55% had cardiovascular failure. Overall, pulmonary compliance and SOFA-R scores improved daily, with a mean change in SOFA-R of −0.7 ± 1.4 and a mean change in dynamic pulmonary compliance of 3.8 ± 18.4 mL/cmH_2_O at 48 h (Table 2). Neither baseline measure of pulmonary dysfunction nor change from baseline to 72 h or change from 24 to 72 h differed across arrest location (data not shown). Patients with an initial arrest rhythm of VT/VF, lower initial PaO_2_:FiO_2_ ratio, and more severe shock were exposed to higher FiO_2_AUCs (Table 3).

**Table 1:** **Baseline population characteristics**

**Characteristic**	**Overall**	**Non-survivors**	**Survivors**
	**(n = 170)**	**(n = 95)**	**(n = 75)**
**Demographics**			
Age, years	60 ± 16	59 ± 17	63 ± 15
Male sex	89 (52%)	54 (61%)	35 (40%)
**Arrest characteristics**			
Out-of-hospital arrest	96 (56%)	58 (61%)	38 (51%)
Initial rhythm VT/VF	64 (38%)	31 (33%)	33 (44%)
Therapeutic hypothermia used	113 (66%)	76 (80%)	37 (49%)
Pittsburgh cardiac arrest category			
1	28 (16%)	9 (9%)	19 (25%)
2	63 (37%)	26 (27%)	37 (49%)
3	32 (19%)	19 (20%)	13 (17%)
4	47 (28%)	41 (43%)	6 (8%)
**Baseline physiologic and ventilator data**			
First PaO_2_:FiO_2_ ratio	241 ± 137	249 ± 129	231 ± 146
Dynamic compliance, mL/cm H_2_O	29 ± 12	29 ± 12	28 ± 13
Initial SOFA-respiratory score			
0	26 (15%)	13 (14%)	13 (17%)
1	33 (19%)	24 (25%)	9 (12%)
2	33 (19%)	17 (18%)	16 (21%)
3	46 (27%)	26 (27%)	20 (27%)
4	32 (19%)	15 (16%)	17 (23%)
Initial SOFA-cardiovascular score			
0	76 (45%)	33 (35%)	43 (57%)
1	18 (11%)	8 (8%)	10 (13%)
2	10 (6%)	9 (9%)	1 (1%)
3	31 (18%)	22 (23%)	9 (12%)
4	25 (21%)	23 (24%)	12 (16%)
Cumulative vasopressor index	1.4 ± 1.9	1.7 ± 1.9	1.1 ± 1.9
Time to first FiO_2_ wean, h	6.3 ± 6.4	6.8 ± 6.9	5.8 ± 5.6
Number of FiO_2_ changes in 24 h	2.5 ± 1.5	2.5 ± 1.6	2.6 ± 1.4

Data are presented as mean (standard deviation) or raw number with corresponding percentage. *VT/VF,* ventricular tachycardia/fibrillation; *PaO*_*2*_ *,* partial pressure of arterial oxygen; *FiO*_*2*_ *,* fraction of inspired oxygen; *SOFA,* sequential organ failure assessment.

**Table 2:** **Population physiological and neurological outcomes**

**Outcome**	**Overall cohort**
	**(n = 170)**
Change in SOFA-respiratory score	
0 to 24 h	−0.3 (1.5)
0 to 48 h	−0.7 (1.4)
0 to 72 h	−0.9 (1.5)
Change in lung compliance, ml/cmH_2_O	
0 to 24 h	0.5 (13.9)
0 to 48 h	3.8 (18.4)
0 to 72 h	4.9 (21.0)
Discharge CPC	
1	8 (5%)
2	1 (1%)
3	58 (34%)
4	7 (4%)
5	94 (56%)
Survival to hospital discharge	75 (44%)

Data are presented as mean (standard deviation) or raw number with corresponding percentage. *SOFA,* sequential organ failure assessment; *CPC,* cerebral performance category.

**Table 3:** **Unadjusted association between oxygen exposure over the first 24 h after ROSC (FIO**_**2**_ **AUC) and baseline arrest and cardiopulmonary characteristics**

**Baseline predictor**	**B coefficient (95% CI)**	***P*** **-value**
Age	−0.035 (−0.069 to −0.001)	0.04
Male sex	0.438 (−0.658 to 1.535)	0.43
Out-of-hospital arrest	0.304 (−0.802 to 1.410)	0.59
Arrest rhythm VT/VF	1.306 (0.191 to 2.421)	0.02
Received TH	0.355 (−0.806 to 1.516)	0.55
PCAC		
1	Reference	Reference
2	−0.246 (−1.823 to 1.332)	0.76
3	2.346 (0.548 to 4.143)	0.01
4	0.441 (−1.217 to 2.100)	0.60
First P:F ratio per 25	−0.262 (−0.354 to −0.170)	<0.001
Initial pulmonary compliance	0.010 (−0.035 to 0.054)	0.67
Initial SOFA-respiratory score		
0	Reference	Reference
1	1.246 (−0.481 to 2.973)	0.16
2	1.703 (−0.024 to 3.430)	0.05
3	3.431 (1.815 to 5.047)	<0.001
4	4.279 (2.540 to 6.018)	<0.001
Initial SOFA-cardiovascular score		
0	Reference	Reference
1	−1.190 (−3.040 to 0.660)	0.21
2	0.145 (−2.230 to 2.519)	0.90
3	1.092 (−2.230 to 2.600)	0.15
4	1.245 (−0.197 to 2.686)	0.09
Initial cardiovascular index	0.346 (0.061 to 0.630)	0.02
Time to first FiO_2_ wean	0.012 (−0.074 to 0.099)	0.78
Number of FiO_2_ changes in 24 h	−0.076 (−0.436 to 0.283)	0.68

*VT/VF,* ventricular tachycardia/ventricular fibrillation; *TH,* therapeutic hypothermia; *PCAC,* Pittsburgh cardiac arrest category; *P:F,* ratio of partial pressure of arterial oxygen (PaO_2_) to fraction of inspired oxygen (FiO_2_); *SOFA,* sequential organ failure assessment.

### Pulmonary dysfunction

In unadjusted analysis, FiO_2_AUC was not significantly associated with increased SOFA-R from 0 to 48 h (Table 4). No baseline predictors were associated with change in dynamic pulmonary compliance from 0 to 48 h (data not shown). In our adjusted model testing independent predictors of change in SOFA-R from 0 to 48 h, FiO_2_ remained non-significant (odds ratio (OR) 0.96 (95% confidence interval (CI) 0.88, 1.04), *P* = 0.34).

**Table 4:** **Unadjusted odds of change in the respiratory subscale of the Sequential Organ Failure Assessment score from 0 to 48 h**

**Predictor**	Unadjusted odds ratio* (95% CI)	***P*** **-value**
FiO_2_ AUC	0.93 (0.86 to 1.02)	0.11
Age	1.00 (0.98 to 1.02)	0.86
Male sex	1.11 (0.62 to 2.00)	0.72
Out-of-hospital arrest	2.02 (1.11 to 3.67)	0.02
Arrest rhythm VT/VF	1.09 (0.78 to 2.00)	0.78
Received TH	2.74 (1.43 to 5.25)	<0.01
PCAC		
1	Reference	Reference
2	2.24 (0.88 to 5.70)	0.09
3	0.87 (0.32 to 2.42)	0.80
4	2.58 (0.97 to 6.84)	0.06
Initial pulmonary compliance	0.98 (0.95 to 1.01)	0.24
Initial SOFA-cardiovascular score		
0	Reference	Reference
1	0.71 (0.27 to 1.85)	0.48
2	1.88 (0.50 to 7.04)	0.35
3	2.11 (0.93 to 4.77)	0.07
4	1.02 (0.47 to 2.24)	0.96
Initial CVI	1.04 (0.90 to 1.20)	0.62
Time to first FiO_2_ wean	0.99 (0.95 to 1.03)	0.67
Number of FiO_2_ changes in 24 h	0.82 (0.68 to 0.99)	0.04

*Odds ratios are presented per unit increase in respiratory subscale score. FiO_2_, fraction of inspired oxygen; AUC, area under the curve; VT/VF, ventricular tachycardia or ventricular fibrillation; TH, therapeutic hypothermia; PCAC, Pittsburgh cardiac arrest category; SOFA, sequential organ failure assessment; CVI, cumulative vasopressor index.

### Secondary outcomes

**Figure 1:**
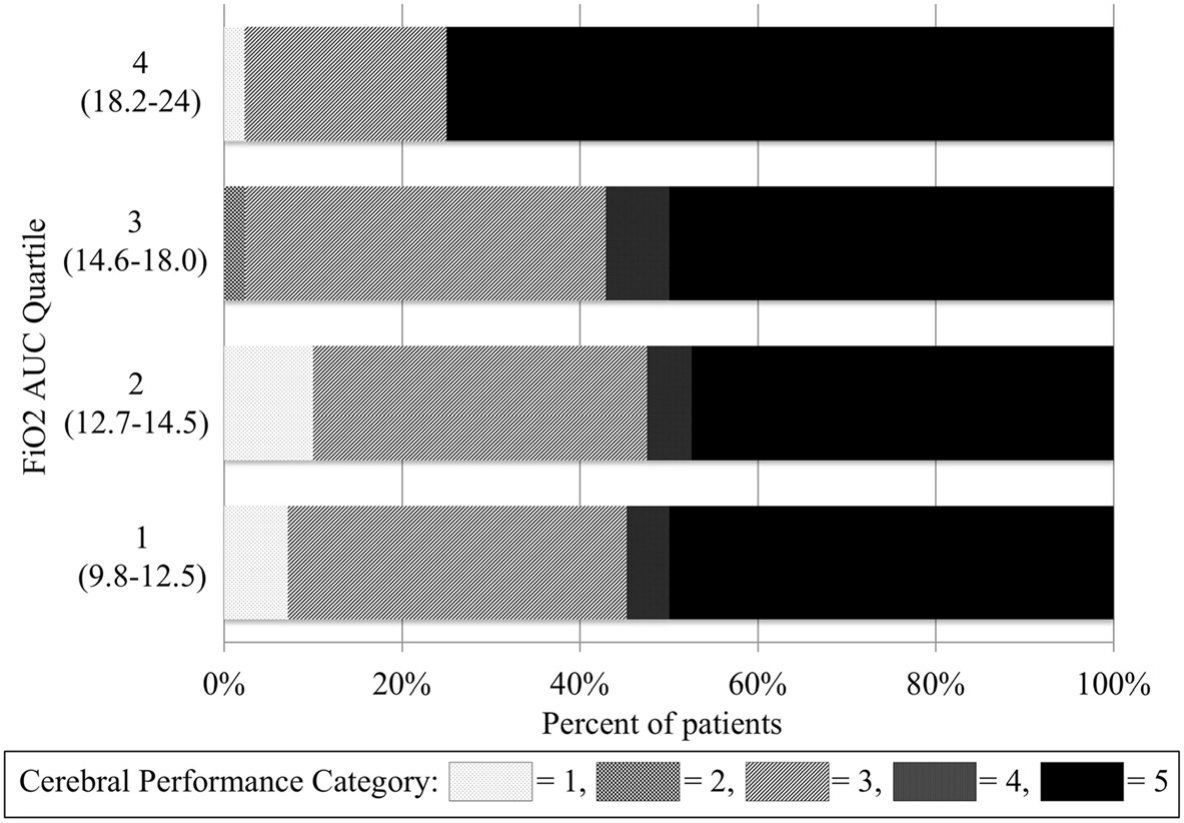
**Cerebral performance category stratified by quartile of fraction of inspired oxygen area under the curve (FiO**_**2**_ **AUC).**

When we tested the association of FiO_2_AUC with outcomes, we found that in both unadjusted and adjusted analysis, higher FiO_2_AUC was associated with significantly lower odds of survival (Table 5) and worse CPC at hospital discharge (Additional file 1: Table S1 and Additional file 2: Table S2). Although our model did not violate the assumption of proportional odds, stratifying patients by FiO_2_AUC quartile revealed that this effect on mortality was driven by lower survival among patients in the highest exposure quartile (OR for survival in the highest quartile compared to the lowest three quartiles 0.32 (95% CI 0.13 to 0.79), *P* = 0.003) (Figure 1).

**Table 5:** **Unadjusted and adjusted associations between predictors and odds of survival to hospital discharge**

**Baseline predictor**	**Odds ratio (OR)**	***P*** **-value**
	**Unadjusted OR (95% CI)**	
FiO_2_ AUC	0.90 (0.82 to 0.98)	0.02
Age	1.02 (1.00 to 1.04)	0.10
Male sex	0.66 (0.19 to 1.22)	0.19
Out-of-hospital arrest	0.66 (0.36 to 1.21)	0.18
Arrest rhythm VT/VF	1.62 (0.87 to 3.03)	0.13
Received TH	0.24 (0.13 to 0.48)	<0.001
PCAC		
1	Reference	Reference
2	0.67 (0.26 to 1.72)	0.41
3	0.32 (0.11 to 0.94)	0.04
4	0.07 (0.02 to 0.22)	<0.001
First P:F ratio	1.00 (1.00 to 1.00)	0.38
Initial pulmonary compliance	0.99 (0.97 to 1.02)	0.68
Initial SOFA-respiratory score		0.23
0	Reference	Reference
1	0.38 (0.13 to 1.11)	0.08
2	0.94 (0.34 to 2.63)	0.91
3	0.77 (0.29 to 2.02)	0.59
4	1.13 (0.40 to 3.19)	0.81
Initial SOFA-cardiovascular score		<0.01
0	Reference	Reference
1	0.95 (0.34 to 2.70)	0.94
2	0.09 (0.01 to 0.71)	0.02
3	0.31 (0.13 to 0.77)	0.01
4	0.40 (0.17 to 0.92)	0.03
Initial CVI	0.82 (0.69 to 0.98)	0.03
Time to first vent wean	1.04 (1.00 to 1.10)	0.08
Number of vent weans in 24 h	0.79 (0.64 to 0.98)	0.03
	**Adjusted OR (95% CI)**	
FiO2 AUC	0.86 (0.76 to 0.97)	0.02
Age	1.01 (0.98 to 1.04)	0.38
Male sex	0.52 (0.23 to 1.18)	0.12
Out-of-hospital arrest	1.48 (0.52 to 4.23)	0.46
Arrest rhythm VT/VF	1.53 (0.62 to 3.77)	0.35
Received TH	0.19 (0.07 to 0.58)	<0.01
PCAC		
1	Reference	Reference
2	1.25 (0.33 to 4.79)	0.74
3	0.91 (0.24 to 3.51)	0.89
4	0.13 (0.03 to 0.58)	<0.01
Initial SOFA-cardiovascular score		
0	Reference	Reference
1	0.62 (0.14 to 2.69)	0.52
2	0.05 (0.00 to 0.71)	0.03
3	0.23 (0.04 to 1.31)	0.10
4	0.12 (0.01 to 1.73)	0.12
Initial CVI	1.29 (0.73 to 2.26)	0.38
Time to first vent wean	0.78 (0.95 to 1.10)	0.13
Number of vent weans in 24 h	1.03 (0.58 to 1.07)	0.49

*FiO*_*2*_, fraction of inspired oxygen; *AU,* area under the curve; *VT/VF,* ventricular tachycardia or ventricular fibrillation; *TH,* therapeutic hypothermia; *PCAC,* Pittsburgh cardiac arrest category; *P:F,* partial pressure of arterial oxygen to fraction of inspired oxygen; *SOFA,* sequential organ failure assessment; *CVI,* cumulative vasopressor index.

### Propensity score-adjusted analysis

The above findings were replicated in our propensity score-adjusted analysis, where only the highest quartile of FiO_2_AUC exposure was associated with increased mortality (*P* = 0.03). The change in SOFA-R and pulmonary compliance from 0 to 48 h did not differ between this quartile and the remaining three quartiles (*P* = 0.18 and *P* = 0.39, respectively). Compared to the other quartiles, the initial SOFA-R in this quartile was significantly higher (median SOFA-R = 3 versus 1, 2 and 2 in quartiles 1 to 3, respectively; *P* <0.001). A propensity-adjusted analysis analyzing the affect of FiO_2_AUC on change in dynamic pulmonary compliance was not possible (terms were not uniquely estimable). In our propensity-adjusted analysis for change in SOFA-R from 0 to 48 h, the highest quartile of FiO_2_AUC was associated with *improved* SOFA-R compared to the lowest quartile (OR 0.41, 95% CI (0.19-0.88), *P* = 0.005) but there were no other significant differences in outcome across quartiles.

## Discussion

We examined the association between inhaled oxygen exposure and change in pulmonary function after CA. Higher oxygen exposure in the initial 24 h was strongly associated with the presence of baseline cardiopulmonary dysfunction, but was not associated with deterioration in pulmonary compliance or gas exchange in the first 48 h after ROSC. In fact, in our propensity-adjusted analysis, CA patients in the highest quartile of oxygen exposure had *improved* lung function compared to the lowest quartile of exposure. Despite the absence of an association with change in lung function, higher inhaled oxygen levels were independently associated with decreased survival and worse neurological outcomes. Taken together, these data suggest that pulmonary oxygen toxicity is not a clinically important mediator of the association between hyperoxia and patient outcomes after CA.

A study of the Project IMPACT database found an association between arterial hyperoxia and worsened survival after CA [8]. The same authors reported increasing odds of mortality in a linear fashion for PaO_2_ values in excess of 100 mmHg, suggestive of dose-dependent toxicity [9]. A significant limitation of this work was the use of a single marker of hyperoxemia (that is, first PaO_2_) without regard to underlying physiologic dysfunction and without assessment of subsequent time points. Furthermore, other large database studies have failed to reproduce this finding [32,33]. Unlike those prior, we have incorporated not only physiologic measures of baseline lung diffusion and cardiac function into our models, expanded our analysis beyond the initial or highest level of oxygenation, and controlled for the appropriate tendency of clinicians to increase FiO_2_ in patients with more severe cardiopulmonary dysfunction.

Our study also differs from previous work in that it examines both surrogate endpoints of pulmonary dysfunction as well as clinically meaningful endpoints. Our results reflect that oxygen exposure was increased in those with the worst early cardiopulmonary dysfunction, which one would expect if oxygen were being titrated based on the clinical assessment of the patient. There was no association between the time to first FiO_2_ wean or the total number of adjustments made in 24 h, suggesting that higher FiO_2_AUC is not a surrogate marker for less attentive care. The correlation between oxygen exposure and SOFA-R, which is based on the PaO_2_:FiO_2_ ratio, is consistent with the appropriate dosing of patients with reduced oxygen diffusive capacity with higher inspired oxygen. The loss of association when comparing early oxygen exposure to subsequent changes in measures of pulmonary dysfunction (SOFA-R or compliance) is contradictory to the notion that early oxygen toxicity results in subsequent lung injury.

Importantly, despite observing no association between oxygen exposure and pulmonary dysfunction, we observed significantly worse survival in the quartile of CA patients with the highest oxygen exposure (25% versus 50%, *P* = 0.003), which remained significant in adjusted analysis and in our propensity-adjusted analysis. This may be explained by the fact that this quartile had the highest baseline SOFA-R scores and may have been sicker than the other quartiles. However, this excess mortality is also consistent with the findings from the Project IMPACT database, although we did not observe a linear dose-response curve as they did. Rather, we observed what appears to be a threshold effect where toxicity accrued only after FiO_2_ exceeded an average of 0.75 over 24 h. If oxidative stress after CA worsens outcomes [34-36] then it is likely that neuronal injury, rather than pulmonary toxicity, drives this effect. Indeed, brain injury is the largest contributor to death after CA [37], though pulmonary dysfunction may play a role, particularly after in-hospital CA [24]. We hypothesize that the threshold for toxicity we observed occurs at the level of exposure at which oxidative stress exceeds antioxidant reserves. Thus, it may be most desirable to decrease FiO_2_ in patients receiving the highest oxygen exposure. Though this would seem intuitive, a recent report revealed that as many as 18% of hyperoxic patients receiving FiO_2_ >0.80 have neither their FiO_2_ nor their PEEP weaned [38].

There are limitations to our study, which are common to retrospective observational studies. Because patients in our study were not randomized, we cannot determine causality. A significant concern in this type of observational study is confounding by indication, whereby sicker patients may also be at higher risk of oxygen exposure, whether appropriate or inappropriate, due to their severity of illness. Propensity-adjusted analysis may partially control for this phenomenon, and our propensity-adjusted analysis for survival replicated our findings. Moreover, in the same propensity-adjusted analysis, those patients in the highest quartile of oxygen exposure had *improved* lung function over 24 h compared to those in the lowest quartile of exposure, further strengthening our assertion that pulmonary oxygen toxicity is not a clinically relevant phenomenon in this patient population. We believe a randomized study would be difficult to perform ethically with the present concerns over hyperoxia after CA, so well-adjusted observational studies are the highest level of evidence possible. Furthermore, our outcome measures (change in SOFA-R and dynamic pulmonary compliance) do not fully represent change in lung dysfunction or the potential for pulmonary oxygen toxicity. There are no compelling data to suggest whether compliance or oxygenation is a better measure of lung dysfunction. As P:F ratio is commonly used in studies of acute lung injury, and previous animal studies have used pulmonary dynamic compliance as a measure of lung function [39,40], we chose to measure both. However, it is important to note that P:F ratio can vary across FiO_2_ irrespective of changes in pulmonary function [41], and post-arrest patients often have significant changes made in FiO_2_ in the first 24 h after ROSC [10]. We believe that we chose the two best measures of lung dysfunction that can be obtained in an observational clinical study, but they are nevertheless imperfect.

Since our work was retrospective, we did not perform a power calculation *a priori,* and unfortunately *post hoc* power calculations have little statistical meaning or utility [42], but our main findings may have resulted from a type II error. Additionally, the observational nature of our study means that our blood gases were not all collected at standardized times and so we were forced to estimate PaO_2_:FiO_2_ ratios and SOFA-R scores using either closely timed blood gases or pulse oximetry data. Further, by limiting our analysis to CA patients who survived and were ventilated ≥24 h, we excluded both the sickest and the healthiest post-arrest patients. Patients who are extubated within 24 h after a CA generally have very good outcomes while withdrawal of care within 24 h is generally due to extremely grim prognosis. By excluding these patients, our analysis may exaggerate the magnitude of the association between oxygen exposure and mortality.

## Conclusion

Our findings suggest that high levels of inhaled oxygen are not associated with deterioration in gas exchange or pulmonary compliance after cardiac arrest. However, oxygen exposure was associated with decreased survival to discharge and worse neurological outcomes. Taken together, it does not appear that pulmonary oxygen toxicity mediates the association between hyperoxia and poor outcomes after cardiac arrest.

## Key messages

- Post-arrest patients are often exposed to 100% oxygen during cardiopulmonary resuscitation and the post-arrest period, raising concern for the potential for pulmonary oxygen toxicity.
- This study suggests that higher oxygen exposure is not associated with subsequent pulmonary dysfunction after cardiac arrest.
- At extreme levels, high oxygen exposure was associated with decreased survival to discharge, probably related to worsened neurological injury.
- We suggest avoiding prolonged exposure to a FiO2 > 0.75 in the early post-arrest period unless necessary to prevent hypoxia.

## Additional files

Additional file 1: Table S1. Unadjusted associations between exposures and discharge cerebral performance category. *Abbreviations*: FiO2 – Fraction of inspired oxygen; AUC – Area under the curve; VT/VF – Ventricular tachycardia or ventricular fibrillation; TH – Therapeutic hypothermia; PCAC – Pittsburgh Cardiac Arrest Category; P:F – Partial pressure of arterial oxygen to fraction of inspired oxygen; SOFA – Sequential Organ Failure Assessment; CVI – Cumulative vasopressor index; OR – Odds ratio. Additional file 2: Table S2. Adjusted associations between exposures and discharge cerebral performance category. Abbreviations: FiO2 – Fraction of inspired oxygen; AUC – Area under the curve; VT/VF – Ventricular tachycardia or ventricular fibrillation; TH – Therapeutic hypothermia; PCAC – Pittsburgh Cardiac Arrest Category; SOFA – Sequential Organ Failure Assessment; CVI – Cumulative vasopressor index; OR – Odds ratio.

## Abbreviations

ARDS: acute respiratory distress syndrome
AUC: area under the curve
CA: cardiac arrest
CPC: Pittsburgh cerebral performance category
CPR: cardiopulmonary resuscitation
CVI: cardiovascular index
FiO2: fraction of inspired oxygen
FiO_2_AUC: area under the curve of the fraction of inspired oxygen for each patient over 24 h
IPTW: inverse probability of treatment weight
MAP: mean arterial pressure
PaO2: partial pressure of arterial oxygen
PCAC: Pittsburgh cardiac arrest category
PCAS: Pittsburgh post-cardiac arrest service
PEA: pulseless electrical activity
PEEP: positive end-expiratory pressure
ROSC: return of spontaneous circulation
SOFA: sequential organ failure assessment score
SOFA-CV: cardiovascular subscale of the sequential organ failure assessment score
SOFA-R: respiratory subscale of the sequential organ failure assessment score
VT/VF: ventricular tachycardia or fibrillation
