# Erratum to: Exposure to high concentrations of inspired oxygen does not worsen lung injury after cardiac arrest

**DOI:** 10.1186/s13054-015-1053-z

**Published:** 2015-09-15

**Authors:** Jonathan Elmer, Bo Wang, Samer Melhem, Raghavesh Pullalarevu, Nishit Vaghasia, Jaya Buddineni, Bedda L. Rosario, Ankur A. Doshi, Clifton W. Callaway, Cameron Dezfulian, on behalf of the University of Pittsburgh Post-Cardiac Arrest Service (PCAS)

**Affiliations:** Safar Center for Resuscitation Research, University of Pittsburgh School of Medicine, 100 Hill Building, 3434 Fifth Avenue, Pittsburgh, 15260 PA USA; Department of Critical Care Medicine, University of Pittsburgh School of Medicine, Pittsburgh, USA; Department of Emergency Medicine, University of Pittsburgh School of Medicine, Pittsburgh, USA; Department of Anesthesiology, New York University Langone Medical Center, Pittsburgh, USA; Department of Internal Medicine, University of Pittsburgh Medical Center Mercy Hospital, Pittsburgh, USA; Department of Epidemiology, Graduate School of Public Health, University of Pittsburgh, Pittsburgh, USA; Vascular Medicine Institute, University of Pittsburgh School of Medicine, Pittsburgh, USA

Unfortunately, the original version of this article [1] contained an error. The spelling of the author Raghavesh Pullalarevu’s name was incorrect. The correct spelling is Raghavesh Pullalarevu rather than “Raghevesh Pullalarevu” which was used in the published article.
