# Supplementary material for: Exposure to high concentrations of inspired oxygen does not worsen lung injury after cardiac arrest
Source: Crit Care. 2015 Mar 10;19(1):105. doi: 10.1186/s13054-015-0824-x (PMC4371614; doi:10.1186/s13054-015-0824-x)
Supplement: Additional file 1: Table S1. — Unadjusted associations between exposures and discharge cerebral performance category. Abbreviations: FiO2 – Fraction of inspired oxygen; AUC – Area under the curve; VT/VF – Ventricular tachycardia or ventricular fibrillation; TH – Therapeutic hypothermia; PCAC – Pittsburgh Cardiac Arrest Category; P:F – Partial pressure of arterial oxygen to fraction of inspired oxygen; SOFA – Sequential Organ Failure Assessment; CVI – Cumulative vasopressor index; OR – Odds ratio. [file 13054_2015_824_MOESM1_ESM.doc]

**Additional file 1: Table S1:** Unadjusted associations between exposures and discharge cerebral performance category

| **Baseline predictor** | **Unadjusted OR (95% CI)** | **P value** |
| --- | --- | --- |
| FiO2 AUC | 1.11 (1.01 – 1.21) | 0.03 |
| Age | 0.99 (0.97 – 1.01) | 0.23 |
| Male sex | 1.32 (0.73 – 2.38) | 0.37 |
| Out-of-hospital arrest | 1.64 (0.92 – 2.89) | 0.10 |
| Arrest rhythm VT/VF | 0.52 (0.28 – 0.97) | 0.04 |
| Received TH | 3.85 (2.02 – 7.33) | <0.001 |
| PCAC |  |  |
| 1 | Ref | Ref |
| 2 | 1.36 (0.58 – 3.18) | 0.48 |
| 3 | 2.55 (0.96 – 6.79) | 0.06 |
| 4 | 16.96 (5.26 – 54.71) | <0.001 |
| First P:F ratio | 1.00 (1.00 – 1.00) | 0.27 |
| Initial pulmonary compliance | 1.00 (0.98 – 1.02) | 0.97 |
| Initial SOFA- Respiratory |  | 0.33 |
| 0 | Ref | Ref |
| 1 | 2.14 (0.74 – 6.23) | 0.16 |
| 2 | 0.93 (0.34 – 2.54) | 0.89 |
| 3 | 0.98 (0.39 – 2.51) | 0.98 |
| 4 | 0.79 (0.29 – 2.11) | 0.63 |
| Initial SOFA- Cardiovascular |  | <0.01 |
| 0 | Ref | Ref |
| 1 | 1.46 (0.53 – 4.02) | 0.47 |
| 2 | 11.52 (1.39 – 95.10) | 0.02 |
| 3 | 2.88 (1.18 – 7.02) | 0.02 |
| 4 | 2.26 (1.02 – 5.00) | 0.05 |
| Initial CVI | 1.17 (0.99 – 1.38) | 0.07 |
| Time to first vent wean | 0.96 (0.92 – 1.00) | 0.06 |
| Number of vent weans in 24h | 1.24 (1.01 – 1.52) | 0.04 |

Abbreviations: FiO2 – Fraction of inspired oxygen; AUC – Area under the curve; VT/VF – Ventricular tachycardia or ventricular fibrillation; TH – Therapeutic hypothermia; PCAC – Pittsburgh Cardiac Arrest Category; P:F – Partial pressure of arterial oxygen to fraction of inspired oxygen; SOFA – Sequential Organ Failure Assessment; CVI – Cumulative vasopressor index; OR – Odds ratio
