# Supplementary material for: Exposure to high concentrations of inspired oxygen does not worsen lung injury after cardiac arrest
Source: Crit Care. 2015 Mar 10;19(1):105. doi: 10.1186/s13054-015-0824-x (PMC4371614; doi:10.1186/s13054-015-0824-x)
Supplement: Additional file 2: Table S2. — Adjusted associations between exposures and discharge cerebral performance category. Abbreviations: FiO2 – Fraction of inspired oxygen; AUC – Area under the curve; VT/VF – Ventricular tachycardia or ventricular fibrillation; TH – Therapeutic hypothermia; PCAC – Pittsburgh Cardiac Arrest Category; SOFA – Sequential Organ Failure Assessment; CVI – Cumulative vasopressor index; OR – Odds ratio. [file 13054_2015_824_MOESM2_ESM.doc]

**Additional file 2: Table S2:** Adjusted associations between exposures and discharge cerebral performance category

| **Baseline predictor** | **Adjusted OR (95% CI)** | **P value** |
| --- | --- | --- |
| FiO2 AUC | 1.16 (1.04 – 1.31) | 0.01 |
| Out-of-hospital arrest | 0.80 (0.32 – 1.97) | 0.62 |
| Arrest rhythm VT/VF | 0.60 (0.27 – 1.34) | 0.22 |
| Received TH | 4.14 (1.59 – 10.81) | <0.01 |
| PCAC |  |  |
| 1 | Ref | Ref |
| 2 | 0.85 (0.28 – 2.57) | 0.77 |
| 3 | 1.00 (0.31 – 3.17) | 1.00 |
| 4 | 8.19 (1.88 – 35.57) | <0.01 |
| Initial SOFA- Cardiovascular |  |  |
| 0 | Ref | Ref |
| 1 | 2.82 (0.77 – 10.34) | 0.12 |
| 2 | 15.27 (1.40 – 266.9) | 0.03 |
| 3 | 3.61 (0.69 – 18.86) | 0.13 |
| 4 | 6.75 (0.62 – 73.0) | 0.12 |
| Initial CVI | 0.80 (0.48 – 135) | 0.41 |
| Number of weans in 24h | 1.35 (1.05 – 1.72) | 0.02 |

Abbreviations: FiO2 – Fraction of inspired oxygen; AUC – Area under the curve; VT/VF – Ventricular tachycardia or ventricular fibrillation; TH – Therapeutic hypothermia; PCAC – Pittsburgh Cardiac Arrest Category; SOFA – Sequential Organ Failure Assessment; CVI – Cumulative vasopressor index; OR – Odds ratio
